# 

**Published:** 1905-04-01

**Authors:** 


### NOTICE TO CORRESPONDENTS.

All MSS., Letters, Books for Review, and other matters intended for the Editor should be addressed The Editor, "The Hospital," The Hospital Buildings, 28 & 29 Southampton Street, Strand, W.C.

The Editor cannot undertake to return rejected MS., even when accompanied by stamped directed envelope.

**Notice to Advertisers and Subscribers.**

All Advertisements, Orders for Copies of The Hospital, and Business Communications should be addressed to **THE MANAGER (Not to the Editor),** The Hospital, 28 & 29 Southampton Street, Strand, London, W.C.

The Cost of Subscription, post free, is at follows:—Per week, 3½d.; per quarter, 3s. 3d.; per half-year, 6s. 6d.; per annum, 13s. Foreign and Colonial:—Per annum, 19s. 6d., payable, in all cases, in advance.

**NOTICE.—Vol. XXXVII. of "The Hospital" October 1904 to March 1905), in handsome cloth binding, will shortly be on sale at the office of this Journal, price 10s. 6d. Cases for binding the Numbers contained in this Volume 2s. Index to Vol. XXXVII., 3½d., post free.**

**The Monthly Part for April is now ready. Price 1s., by post, 1s. 3d.**

### Medical Appointments.

George Basil Adams, M.B., B.S.Oxon., Resident Medical Officer at the City of London Hospital for Diseases of the Chest, Victoria Park, E. **John Parkinson Atkinson**, M.D.Glasg., L.R.C.P.Lond., L.R.C.S.Edin., Medical Officer of Health of Lynton. **S. L. Benton**, M.R.C.S., L.R.C.P.Lond., House Physician to the City of London Hospital for Diseases of the Chest, Victoria Park, E. **Frederick Charles Berry**, M.D., B.Ch.Dub., reappointed Medical Officer of Health of Burnham (Somerset). **A. H. Brewer**, M.R.C.S., L.R.C.P.Lond., Honorary Anæsthetist to the Metropolitan Throat and Ear Hospital. **E. Farquhar Buzzard**, M.D.Oxon., M.R.C.P.Lond., Assistant Physician to the National Hospital for the Paralysed and Epileptic, Queen Square, London, W.C. **John E. Chapman**, M.R.C.S., L.R.C.P.Lond., District Medical Officer by the South Molton (Devon) Board of Guardians. **Lucy B. Clapham**, M.B.Lond., Inspector under the Central Midwives Board. **Carey Franklin Coombs**, M.D., B.S.Lond., Curator of the Museum at the Bristol General Hospital. **William Dick**, L.R.C.P. & S.Edin., Junior House Surgeon to the General Infirmary, Macclesfield. **J. P. Drinkwater**, M.R.C.S.Eng., reappointed Medical Officer to the Llangollen Urban District Council. **O. F. F. Grunbaum**, M.D.Cantab., M.R.C.P.Lond., D.Sc., Physician to Out-patients at the City of London Hospital for Diseases of the Chest, Victoria Park, E. **Edgar Rastricke Hanson**, L.R.C.P. & S.Edin., District Medical Officer by the South Molton (Devon) Board of Guardians. **Alfred Harvey**, M.B.Lond., M.R.C.S., L.S.A., Medical Officer to the Children's Homes by the Bristol Board of Guardians. **Charles Vernon Hitchens**, M.R.C.S., L.S.A., reappointed Medical Officer of Health of Weston-super-Mare. **H. M. Joseph**, M.B., Resident Medical Officer at the Hospital for Epilepsy and Paralysis, Maida Vale. **Stuart A. Moore**, B.A., M.B., B.Ch.Edin., Junior House Surgeon to the Liverpool Stanley Hospital. **Albert T. Morgad**, M.D.Brux., L.S.A., Medical Officer to the Children's Homes by the Bristol Board of Guardians. **W. Laidlaw Purves**, M.D.Edin., M.R.C.S., Consulting Ophthalmic Surgeon to the Hospital for Epilepsy and Paralysis, Maida Vale. **George W. Ross**, M.R.C.S., L.R.C.P.Lond., House Physician to the City of London Hospital for Diseases of the Chest, Victoria Park, E. **H. James Smyth**, M.R.C.S., L.R.C.P.Lond., District Medical Officer by the South Molton (Devon) Board of Guardians. **George F. Sydenham**, M.R.C.S., L.S.A., District Medical Officer by the South Molton (Devon) Board of Guardians. **George Christopher Tayler**, M.D., L.R.C.P.Lond., M.R.C.S., Medical Officer for the Third District by the Westbury (Wiltshire) Board of Guardians. **E. J. Tongue**, M.R.C.S., L.R.C.P.Lond., Certifying Surgeon under the Factory and Workshop Act for the Winterton District of the county of Lincoln. **Reginald Wade**, M.R.C.S., L.S.A., reappointed Medical Officer of Health of the Highbridge (Somerset) Urban District. **W. Harper Wigham**, M.D.Durh., M.R.C.S., District Medical Officer by the South Molton (Devon) Board of Guardians. **Nicholas Vincent Wise**, L.R.C.P. & S.Irel., Medical Officer for the Second District by the Westbury (Wiltshire) Board of Guardians. **James Young**, M.D., C.M.Glasg., Medical Officer to the Children's Homes by the Bristol Board of Guardians.

"The Hospital" with its Nursing Section supplies the comprehensive information on medical, institutional and nursing matters which alone can satisfy the intelligently progressive nurse of to-day.

The majority of nurses who are attached to institutions are in a position to see regularly "The Hospital" in the reading rooms and clubs, whether they take it individually or not. "The Hospital," therefore, in its present form meets the needs of a large number of working nurses throughout the Empire, to whom it has in fact become a necessity.

### Important Announcement

### A NEW DEPARTURE.

There are, however, a large number of nurses who feel the need of

**a Nursing Paper at a Penny,**

which they can always keep by them. In response to the repeated representations that have been received from nurses, it has therefore been decided to publish the Nursing Section of "The Hospital" on and after April 8 separately at

### ONE PENNY WEEKLY.

This separate Issue will be entitled

### "THE NURSING MIRROR,"

and will, for the present at any rate, contain the same literary matter, advertisements, situations vacant, wanted, &c., as "The Hospital" Nursing Section, which **WILL STILL BE ISSUED WITH** "The Hospital."

### "THE NURSING MIRROR"

will be published each week as a separate paper, and will comprehend all those advantages which long establishment and intimate relations with the nursing world can alone command.

### "THE NURSING MIRROR"

will be published on Friday, and will be obtainable at this Office, and from all Booksellers and Newsagents on that day.

Nurses desiring copies of "THE NURSING MIRROR" should advise their Newsagents without delay.

"The Nursing Mirror," "The Hospital" Building, 28 & 29 Southampton Street, Strand, London, W.C.

